# Isolation of Apigenin from Sungkai ( *Peronema canescens*) Leaves and Its Immunomodulatory Effects: An In Vivo Study on Granzyme B, Interferon-γ, and Perforin Expression with Supporting In Silico Analysis

**DOI:** 10.12688/f1000research.167153.1

**Published:** 2025-08-08

**Authors:** Dwisari Dillasamola, Yufri Aldi, Najmiatul Fitria, Biomechy Oktomalioputri, Uce Lestari, Risma Multia

**Affiliations:** 1 Pharmacy, Universitas Andalas, Padang, West Sumatra, Indonesia; 2 Medicine, Universitas Andalas, Padang, West Sumatra, Indonesia; 3 Medicine, Universitas Jambi, Jambi, Jambi, Indonesia

**Keywords:** Apigenin, granzyme, immunomodulatory, interferon-gamma, sungkai.

## Abstract

**Background:**

*Paronema canescens* Jack., commonly known as sungkai, is a medicinal plant native to Southeast Asia, particularly abundant in the forests of Sumatra and Borneo, Indonesia. Traditionally, sungkai has been used to treat various ailments, likely due to its rich content of bioactive secondary metabolites. This study aimed to isolate, characterize, and evaluate the immunostimulatory and anti-inflammatory potential of compounds from sungkai leaves based on in silico and in vivo analyses.

**Methods:**

Apigenin was isolated from *Paronema canescens* leaves via ethanol extraction, liquid-liquid partitioning, and chromatographic purification, then characterized by UV-Vis, FT-IR, and NMR spectroscopy. Molecular docking was conducted using MOE software to assess apigenin’s binding to granzyme B, perforin, and IFN-γ, with levamisole as a reference. In vivo, 25 male mice were randomized into five groups and administered apigenin (1, 25, or 50 mg/kg BW) intramuscularly for seven days, alongside COVID-19 vaccination. Granzyme B and IFN-γ serum levels were quantified using ELISA. Statistical analysis employed one-way ANOVA with Duncan’s test ( *p* < 0.05).

**Results:**

The *in silico* analysis demonstrated that apigenin exhibited favorable binding affinities and multiple stabilizing interactions with granzyme B, perforin, and interferon-γ, supporting its potential role in enhancing cellular immune responses through direct molecular modulation of key cytotoxic effector proteins. To assess its immunostimulatory activity in vivo, apigenin was orally administered to mice ( *Mus musculus*) at doses of 1, 25, and 50 mg/kg body weight. Mice were pre-induced with a COVID-19 vaccine to simulate immune system activation. Immunological responses were evaluated through the measurement of granzyme B, perforin, and interferon-γ expression levels. The results demonstrated that apigenin significantly increased the expression of all three markers in a dose-dependent manner.

**Conclusion:**

Collectively, the chemical, computational, and biological data confirm that apigenin from sungkai leaves holds strong immunostimulant and selective anti-inflammatory potential, supporting its development as a natural immune booster or vaccine adjuvant.

## Introduction

Indonesia, as a tropical country with extensive rainforest ecosystems, harbors one of the highest levels of biodiversity in the world. ^1– 3^ These forests support numerous ecological functions, such as nutrient cycling, energy flow, prevention of soil erosion, and facilitation of photosynthesis. ^4, 5^ However, the vast array of plant species within these ecosystems remains underexplored for their medicinal potential. ^6, 7^ One such underutilized plant is *Paronema canescens* Jack., commonly known as sungkai, a member of the Lamiaceae family. Sungkai is widely cultivated by local communities and naturally grows in forests, gardens, and residential areas. ^8^ Ethnobotanical records indicate that this plant has traditionally been used in Sumatra and Borneo to treat various ailments, including malaria, fever, hypertension, intestinal worm infections, and hypercholesterolemia. ^8– 10^ Phytochemical analyses have identified the presence of flavonoids, alkaloids, and terpenoids in sungkai leaves. ^11– 13^

Previous studies have successfully isolated apigenin, a major flavonoid from sungkai leaves, which demonstrated significant anti-inflammatory activity. ^14^ Apigenin, a polyphenolic compound, has been reported to exhibit a wide range of pharmacological properties, including antioxidant, anti-inflammatory, antiviral, and immunomodulatory effects, primarily due to its phenolic structure.

The immune system plays a crucial role in protecting the body from infectious agents such as viruses. Impaired immune responses, which may be exacerbated by psychological stress and social isolation, can increase susceptibility to infections. ^15– 18^ Enhancing immune function is a key preventive strategy. ^19, 20^

Granzyme B (GzmB), produced by cytotoxic T lymphocytes and natural killer (NK) cells, is an essential effector molecule that promotes the destruction of virus-infected or malignant cells. ^21, 22^ GzmB is also known to cleave viral proteins involved in replication, such as those of the herpes simplex virus. ^23^

While compounds such as stigmasterol and bis(2-ethylhexyl) phthalate from sungkai have shown immunostimulatory effects by increasing NK cell levels, ^24^ the potential of apigenin as an immunostimulant remains unexplored. Given apigenin’s broad pharmacological profile and its presence as a major constituent of sungkai leaves, it is imperative to investigate its possible role in enhancing immune responses. Therefore, this study aims to evaluate the immunostimulatory activity of apigenin isolated from sungkai leaves by assessing its effects on key immune markers (Granzyme B, Interferon-gamma (IFN-γ), and Perforin) in a murine model.

## Methods

### Chemicals, reagents, and instruments

Analytical procedures utilized a variety of laboratory instruments and materials. Weighing was conducted using an analytical scale (Ohaus ^®^), while absorbance readings were obtained via a UV–Vis spectrophotometer (Beckman Coulter ^®^). Chromatographic separation was performed using radial chromatography with silica gel 60 PF254 containing gypsum (Merck, Cat. No. 1.07749.1000). For TLC profiling, aluminum-backed silica gel 60 F254 plates (Merck, Cat. No. 1.05554.0001; 0.25 mm thickness) were used and visualized under 254 nm UV light or after spraying with 10% H _2_SO _4_ (v/v) in methanol.

Advanced compound identification was conducted using a Vanquish™ UHPLC Binary Pump (Thermo Scientific™, Germany) coupled to a Q Exactive™ Hybrid Quadrupole-Orbitrap™ Mass Spectrometer. Separation was achieved using a Thermo Scientific™ Accucore™ Phenyl-Hexyl column (100 mm × 2.1 mm ID × 2.6 μm). Solvents used for extraction, chromatography, and sample preparation included methanol (MeOH; Merck, Cat. No. 106009), ethanol (EtOH; Merck, Cat. No. 100983), ethyl acetate (EtOAc; Merck, Cat. No. 109623), and Milli-Q water (Merck Millipore). Additional laboratory tools comprised Pyrex ^®^ beakers, droppers, probes (Terumo ^®^), microscope (Olympus ^®^), ELISA microplate reader (Thermo Scientific™ Multiskan), and standard laboratory glassware.

Reagents used in animal treatments included: Sodium acetate 1 M (Merck, Cat. No. 106268), Sodium carboxymethyl cellulose (NaCMC) 0.5% (Sigma-Aldrich, Cat. No. C4888) as a suspension vehicle, Aqua pro injection (Andeska Lab, Indonesia, Cat. No. AQPRO-INJ-500), Aquadest (Andeska Lab, Cat. No. AQ-DES-1000), COVID-19 vaccine (Indovac ^®^, Bio Farma, Indonesia).

Biological materials included male white mice ( *Mus musculus*, 6–8 weeks old, 25–30 g) from PT Indoanilab (Indonesia), standard mouse pellet diet (Vita Feed ^®^), and apigenin isolated from *Peronema canescens* leaves.

### Plant materials

Leaves of *Paronema canescens* Jack. (sungkai) were collected from Aia Pacah, Padang City, West Sumatera, Indonesia. The plant was identified and authenticated by Dr. Nurainas at the Herbarium ANDA, Department of Biology, Universitas Andalas, with voucher specimen number 717/K-ID/ANDA/X/2024. Samples were air-dried and ground to a fine powder before extraction.

### Extraction

Seventeen kilograms of dried sungkai leaf powder were macerated in 96% ethanol (2:5 w/v) at room temperature for 72 hours. The extraction process was repeated every 24 hours by decanting the extract and re-macerating the residue. The combined filtrates were concentrated using a rotary evaporator to yield approximately 1.2 kg of crude extract.

***Isolation and characterization***

Five hundred grams of the concentrated extract were fractionated via liquid-liquid extraction using increasing polarity solvents: *n*-hexane, ethyl acetate, and ethanol. The ethanol-soluble fraction was further subjected to vacuum liquid chromatography using gradients of *n*-hexane: EtOAc (100:0 to 0:100) and methanol. This yielded 14 initial fractions, which were combined based on TLC spot profiles into five main fractions.

Fraction X (FX), displaying prominent UV-active spots, was purified using radial chromatography with silica gel-coated plates (0.5 mm thickness) and solvent gradients of *n*-hexane: EtOAc (6:4, 5:5, 3:7). Approximately 10 g of a yellow solid (Isolate 1) was obtained.

Spectroscopic analyses identified Isolate 1 as apigenin ( Table 1), based on the following data:

• UV-Vis (MeOH): λ_max 210, 267, and 336 nm
• FT-IR: ν_OH 3286 cm ^−1^; ν_C=O 1653 cm ^−1^; ν_C=C aromatic 1587–1446 cm ^−1^; ν_C-O 1298–1031 cm ^−1^
• ^1^H-NMR and ^13^C-NMR (DMSO-d _6_, 500/125 MHz): Confirmed the flavonoid structure with corresponding HMBC and COSY correlations.

**Table 1.** MS (m/z 268.992 [M-H] ^-^) for C _15_H _10_O _5_.

No	DEPT-135°	^13^C (ppm) *	HSQC ( *mult.*, *J* in Hz, ΣH) **	HMBC	COSY
2	C	164.2	-	-	-
3	CH	102.9	6.78 ( *s*, 1H)	C-2, C-4, C-10, C-1'	-
4	C	181.8	-	-	-
5	C	161.5	-	-	-
6	CH	98.9	6.18 ( *d*, *J* = 2.05 Hz, 1H)	C-5, C-7, C-8, C-10	-
7	C	163.8	-	-	-
8	CH	94.0	6.47 ( *d*, *J* = 2.1 Hz, 1H)	C-6, C-7, C-9, C-10	-
9	C	157.3	-	-	-
10	C	103.7	-	-	-
1'	C	121.2	-	-	-
2', 6'	CH	128.5	7.92 ( *d*, *J* = 8.75 Hz, 2H)	C-2, C-4', C-3', 5'	H-3', 5'
3', 5'	CH	116.0	6.92 ( *d*, *J* = 8.75 Hz, 2H)	C-1', C-4', C-2', 6'	H-2', 6'
4'	C	161.2	-	-	-

** (DMSO-d6; 1H-NMR 500 MHz; 13C-NMR 125 MHz).

* (DMSO-d6; 13C-NMR 125 MHz).

### In Silico study

***Ligand and protein preparation***

The 3D structure of apigenin (CID: 5280443) and Levamisole (CID: 26879) was retrieved from the PubChem website ( https://pubchem.ncbi.nlm.nih.gov/). Crystal structures of granzyme B (PDB: 1FQ3), perforin (PDB: 3NSJ), and interferon-γ (PDB: 1FG9) were retrieved from the Protein Data Bank ( https://www.rcsb.org/). Protein and ligand were optimized with MOE 2022.02 (licensed proprietary software, license was obtained) to a gradient convergence of 0.001 kcal/mol/Å ^2^.

***Molecular docking***

The active sites of each target protein were identified using the Site Finder tool in MOE. The Molecular docking simulations were carried out using the default settings of the MOE applications. Ligand–protein interactions were evaluated based on binding affinity (kcal/mol); more negative binding affinities were considered to exhibit favorable binding stability and orientation. Levamisole, a known immunomodulatory agent, was used as a reference compound for comparison. Following docking, ligand–protein interactions were analyzed through 2D and 3D visualizations using the Biovia Discovery 2022 application.

### In vivo assay of expression of Granzyme B

***Experimental design***

This study was a randomized controlled in vivo study conducted to evaluate the immunostimulatory effect of apigenin, isolated from *Paronema canescens* (sungkai) leaves, in male mice immunized with the COVID-19 vaccine. This study utilized 25 healthy mice ( *Mus musculus*), each weighing between 25 and 40 grams. Only healthy male mice aged 6–8 weeks and weighing between 25 and 40 grams were included. Mice were obtained from a certified vendor and allowed a 7-day acclimatization period before treatment. Mice showing signs of illness, injury, or abnormal behavior during the acclimatization period were excluded. No animals were excluded after allocation, and all animals that began treatment completed the protocol and were included in the final analysis.

The animals were randomly assigned to five groups (n = 5 per group) as follows:

• Group 1 (Negative control): Received only the drug carrier, 0.5% sodium carboxymethyl cellulose (NaCMC).
• Group 2 (Positive control): Received the COVID-19 vaccine along with 0.5% NaCMC as the carrier.
• Group 3 (Apigenin 1 mg/kg BW): Received the COVID-19 vaccine and apigenin at a dose of 1 mg/kg body weight, formulated in 0.5% NaCMC.
• Group 4 (Apigenin 25 mg/kg BW): Received the COVID-19 vaccine and apigenin at a dose of 25 mg/kg body weight, formulated in 0.5% NaCMC.
• Group 5 (Apigenin 50 mg/kg BW): Received the COVID-19 vaccine and apigenin at a dose of 50 mg/kg body weight, formulated in 0.5% NaCMC.

All mice underwent a 7-day acclimatization period to allow adaptation to the laboratory environment and reduce stress-related variability. On Day 1, all groups except the negative control (Group 1) were immunized with the COVID-19 vaccine. Apigenin was administered intramuscularly once daily for 7 consecutive days (Days 1–7) to Groups 3, 4, and 5 according to their respective dosages. On Day 8, a booster dose of the COVID-19 vaccine was administered to all mice in Groups 2 through 5. On Day 9, before blood collection, animals were sedated using diethyl ether as an inhalation anesthetic. The anesthetic was administered by placing a cotton pad soaked in diethyl ether into a sealed treatment chamber. Each mouse was then gently placed into the chamber and monitored until sedation was achieved. Standard equipment used during animal procedures included feeding sonde, sterile disposable syringes, and surgical blades (scalpels). Blood samples were collected following euthanasia by guillotine decapitation targeting the carotid artery. This method was selected to ensure rapid and humane termination under deep sedation, minimizing animal distress and ensuring optimal blood volume recovery for downstream analyses. All procedures were performed by trained personnel under the supervision of a laboratory veterinarian to ensure animal welfare and procedural consistency. After blood collection, the samples were allowed to clot at room temperature and subsequently centrifuged to separate the serum. The isolated serum was aliquoted and stored under appropriate conditions until further analysis.

***Granzyme B expression assay***

The level of granzyme B was quantified using an ELISA kit (BT Lab ^®^, Cat. No. E0846Mo) according to the manufacturer’s instructions. Standards were prepared at concentrations of 1600, 800, 400, 200, 100, 50, and 0 pg/mL. Each well received 50 μL of the granzyme B standard or sample, followed by 50 μL of detection antibody. The plate was incubated at room temperature (25°C) for 1 hour, then washed three times with 350 μL of wash buffer (BT Lab ^®^, provided in the kit). After washing, 100 μL of TMB substrate (BT Lab ^®^, supplied with the kit) was added per well and incubated in the dark for 10 minutes. The reaction was stopped with 100 μL of stop solution (BT Lab ^®^), and absorbance was read at 450 nm using a microplate reader. Granzyme B concentrations were calculated based on the standard curve generated from known concentrations.

***Interferon gamma level examination***

Serum IFN-γ levels were measured using a commercial ELISA kit (BT Lab ^®^, Cat. No. E0002Mo) according to the manufacturer’s instructions. Each well was loaded with 50 μL of serum or standard, followed by antibody incubation and washing steps as recommended. Subsequently, 100 μL of TMB substrate was added to initiate the enzymatic reaction, followed by 10 minutes of incubation. The reaction was stopped with 100 μL of stop solution, and absorbance was measured at 450 nm. The concentrations were derived from a standard curve generated using IFN-γ standards provided in the kit.

***Statistical analysis***

All data were presented as mean ± standard deviation (SD). Differences between groups were analyzed using one-way analysis of variance (ANOVA). Post hoc comparisons were performed using Duncan’s multiple range test to determine statistically significant differences among groups. Statistical significance was defined as p < 0.05. All analyses were carried out using SPSS Statistics version 15.0 (SPSS Inc., Chicago, IL, USA).

### Ethical considerations

All animal procedures were conducted following the ethical standards for animal experimentation and complied with institutional and national guidelines. Ethical approval for this study was obtained from the Health Research Ethics Committee of the Faculty of Pharmacy, Andalas University, under approval number: 93/UN16.10.D.KEPK-FF/2024. All efforts were made to minimize animal suffering, and only the minimum number of animals required to achieve statistical significance was used. Sedation and euthanasia procedures were performed to ensure humane treatment throughout the experimental protocol.

## Results

### Compounds isolations

Isolate 1 was successfully obtained from fraction FX of the sungkai leaf extract, yielding 10 grams of yellow powder solid that was soluble in ethanol. The TLC profile showed a single spot under UV light at 254 and 365 nm, indicating the presence of a single compound.

### UV–Visible spectroscopy (UV–Vis)

**Figure 1.**
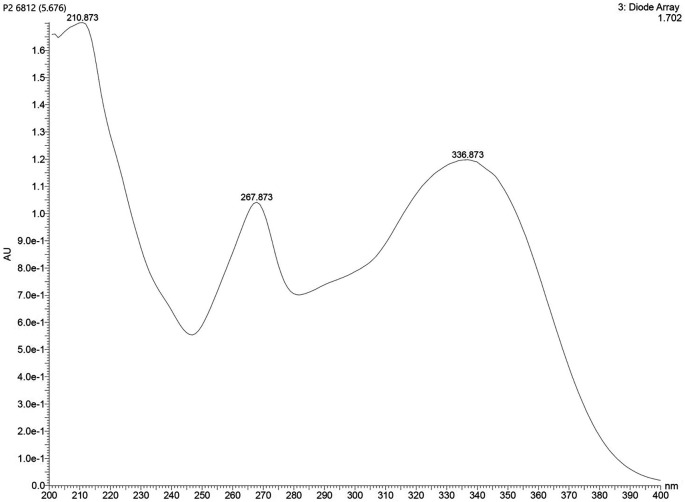
Spectrum UV-Vis isolate 1.

The UV spectrum of Isolate 1 exhibited three maximum absorption peaks at λmax 210, 267, and 336 nm. These peaks suggested the presence of a conjugated chromophore system ( Figure 1).

### Fourier-Transform Infrared Spectroscopy (FTIR)

**Figure 2.**
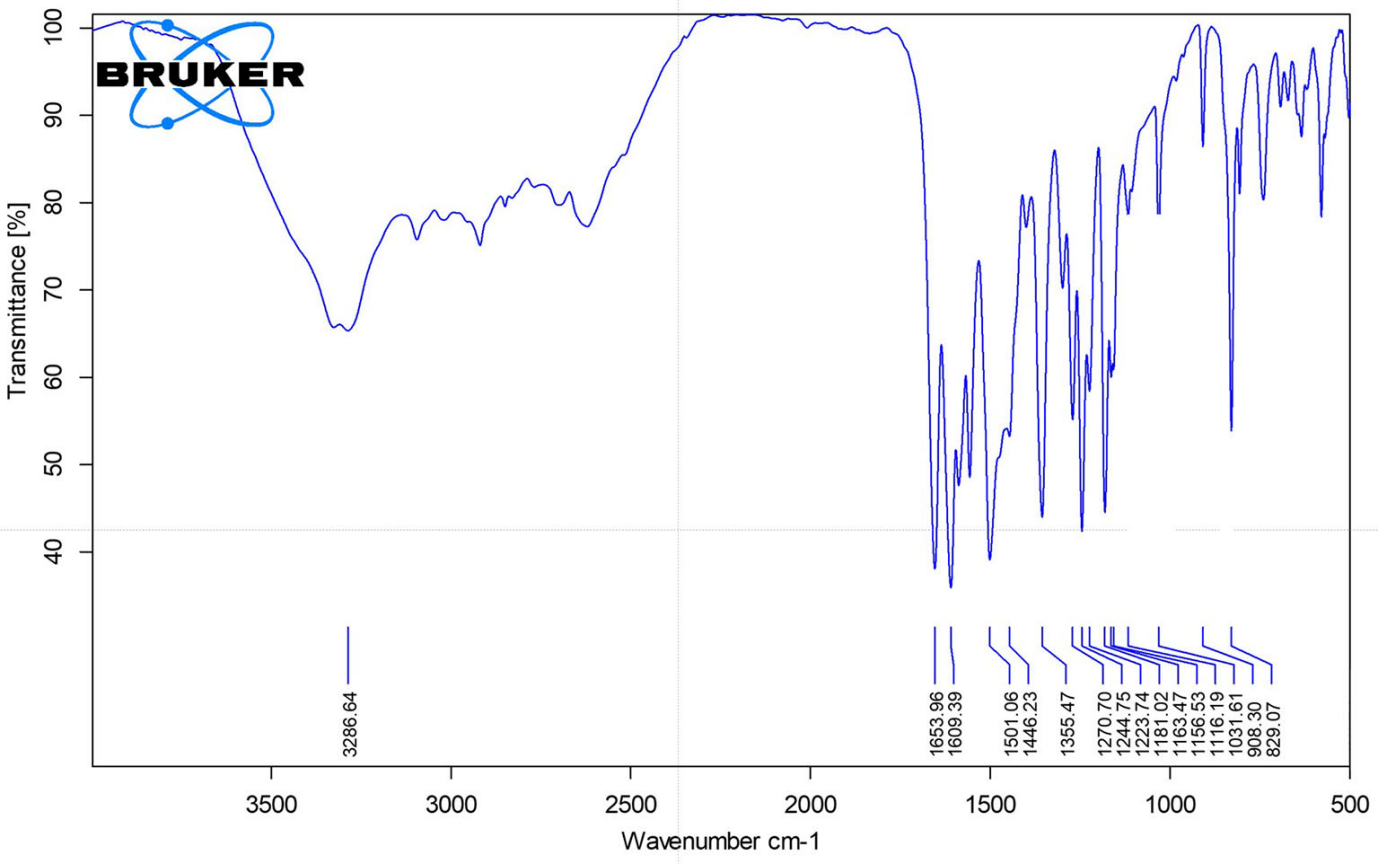
Spectrum FT-IR isolate 1.

FTIR spectrum showed characteristic peaks at Vmax 3286 cm ^−1^ (O–H stretch), 1653 cm ^−1^ (C=O stretch), 1587–1446 cm ^−1^ (aromatic C=C), and 1298–1031 cm ^−1^ (C–O stretch), indicating the presence of hydroxyl, carbonyl, and aromatic functional groups ( Figure 2).

### Proton Nuclear Magnetic Resonance ( ^1^H-NMR)

**Figure 3.**
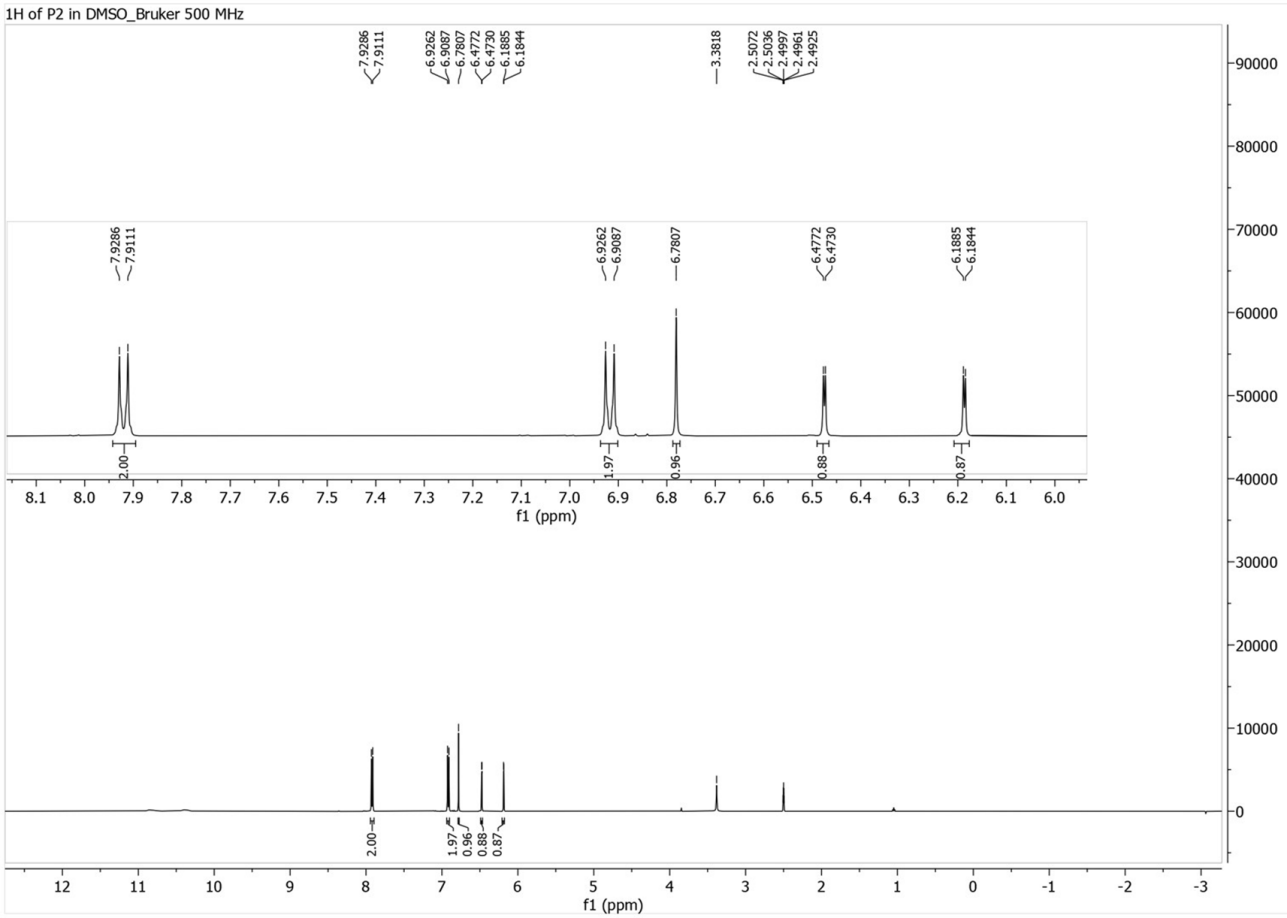
^1^H NMR spectrum for isolate 1 in DMSO-d6 (500 MHz).

The ^1^H-NMR spectrum revealed five proton signals in the δH 6–8 ppm region. Signals at δH 7.92 and 6.92 ppm appeared as doublets with J = 8.75 Hz, suggesting ortho-coupled aromatic protons. Signals at δH 6.47 and 6.18 ppm showed meta coupling (J ≈ 2 Hz), while a singlet at δH 6.78 ppm was attributed to an isolated proton between two quaternary carbons ( Figure 3).

### Carbon-13 NMR ( ^13^C-NMR)

**Figure 4.**
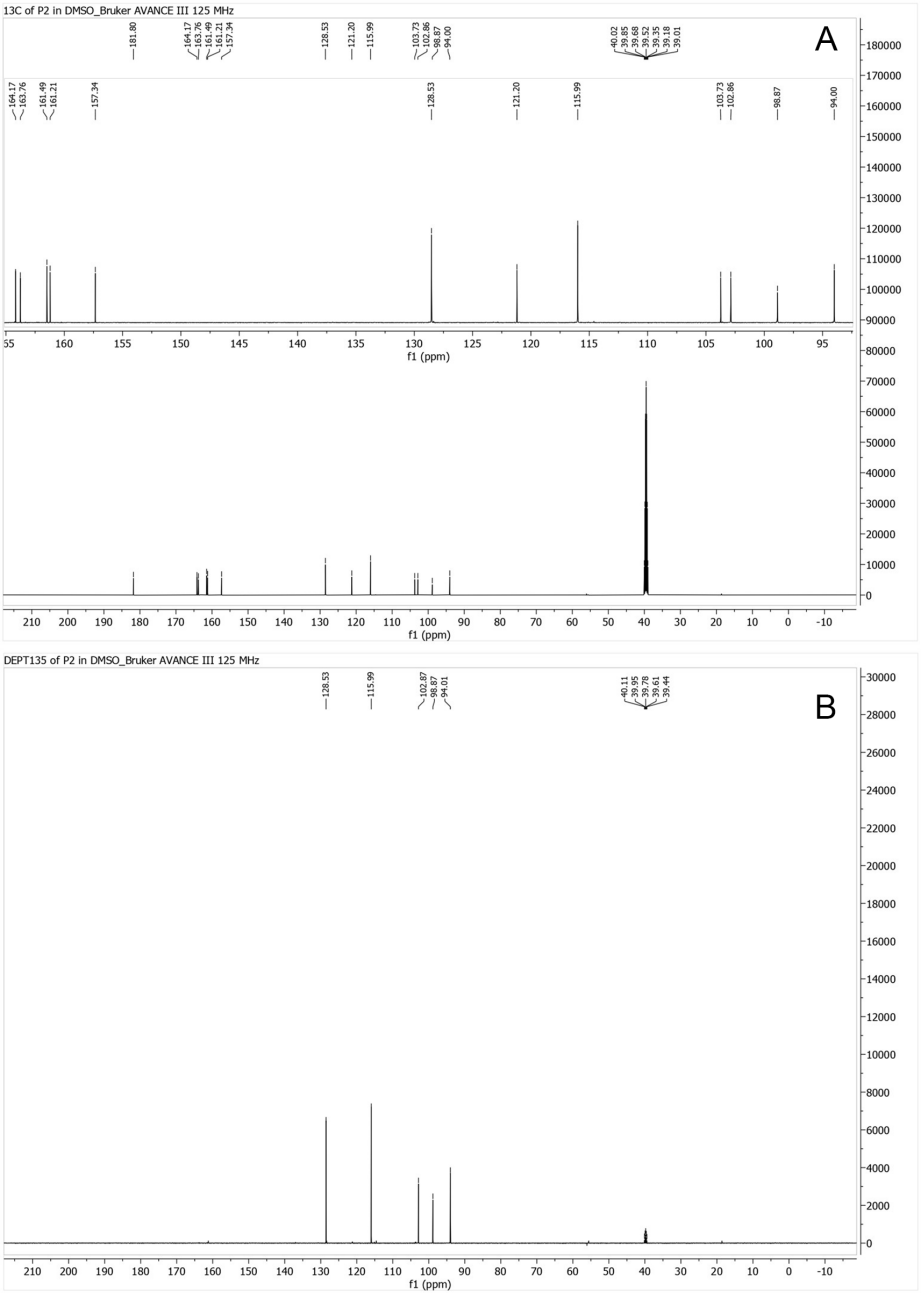
^13^C NMR spectrum for isolate 1 in DMSO-d6 (125 MHz).

The ^13^C-NMR spectrum exhibited 13 carbon signals, all in the sp ^2^ region. Six signals between δC 157.3–181.8 ppm corresponded to =C–OH and –C=O carbons, while the remaining seven signals (δC 94.0–128.5 ppm) represented sp ^2^ methine and quaternary carbons ( Figure 4).

### COSY spectrum

**Figure 5.**
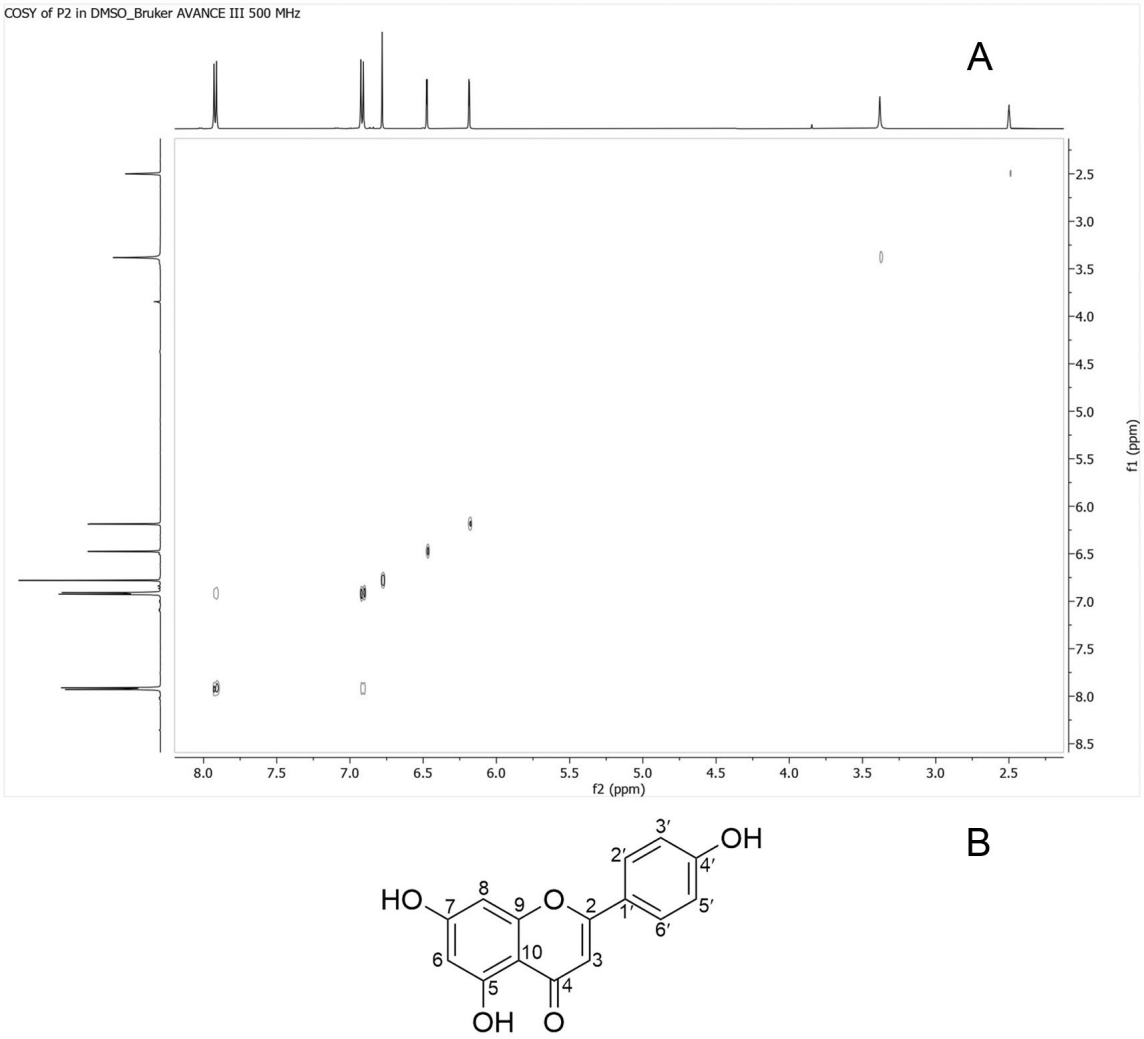
COSY spectrum of isolate 1.

COSY analysis confirmed proton-proton correlations, notably between δH 7.92 and 6.92 ppm, supporting ortho substitution patterns. No direct coupling was observed among the remaining three protons ( Figure 5).

### HMBC spectrum

**Figure 6.**
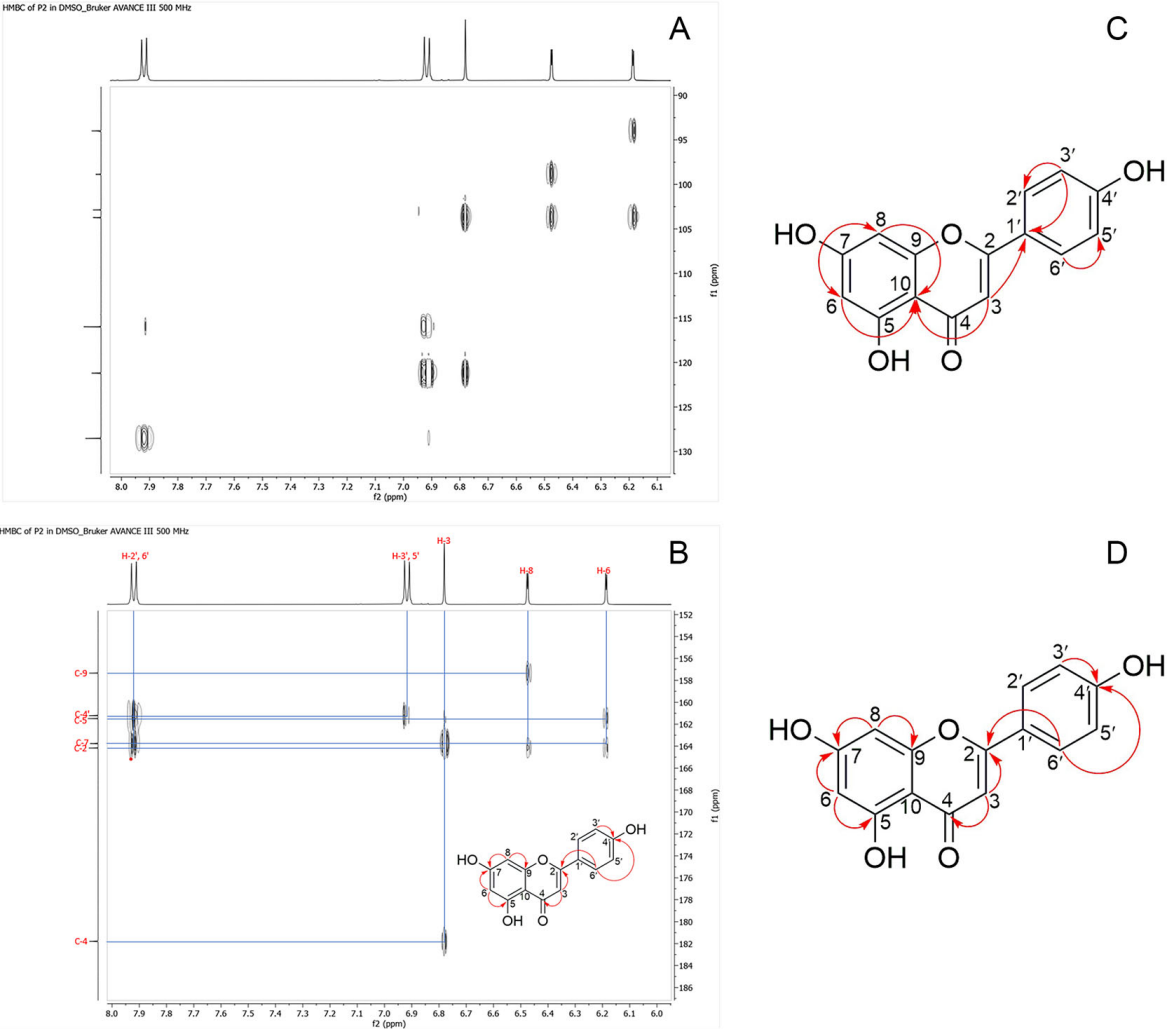
HMBC (Heteronuclear Multiple Bond Correlation) spectrum of isolate 1.

Long-range correlations observed in the HMBC spectrum confirmed the positioning of key functional groups. δH 6.78 ppm correlated with C-2, C-4, C-10, and C-1’, indicating carbonyl at C-4 and phenolic group at C-2. δH 6.18 and 6.47 ppm showed meta correlations to C-5, C-6, C-7, C-8, C-9, and C-10, confirming OH substitution at C-5 and C-7 ( Figure 6).

### Mass Spectrometry (MS)

**Figure 7.**
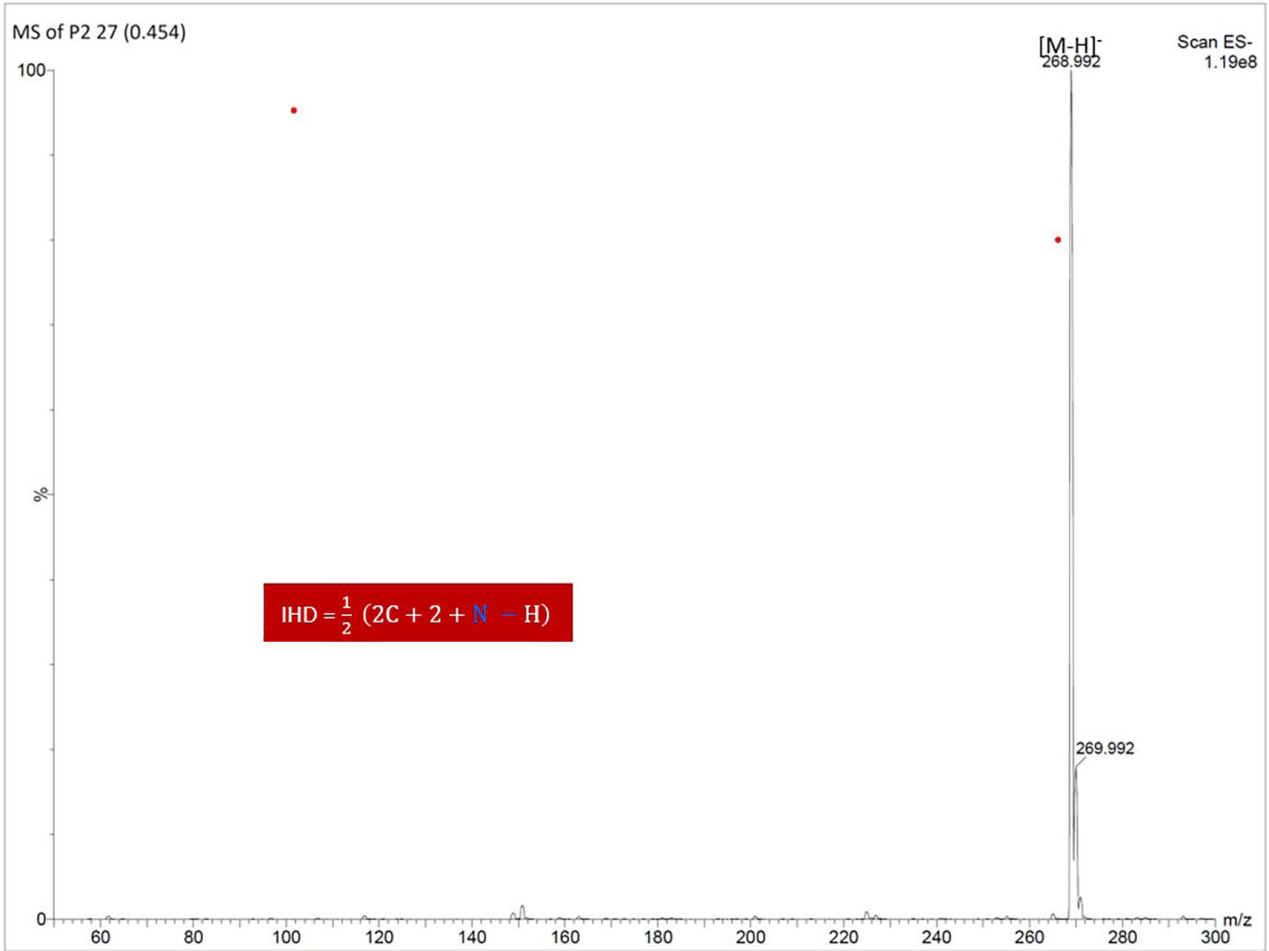
Mass spectrum of isolate 1 Using (-) mode.

Using UPLC-MS/MS in negative ion mode, the molecular ion peak [M–H] ^-^ was detected at m/z 268.992, corresponding to the molecular formula C _15_H _10_O _5_. This confirmed the identity of the compound as apigenin ( Figure 7).

### Index of Hydrogen Deficiency (IHD)

**Figure 8.**
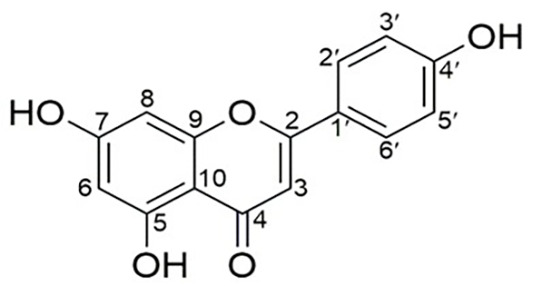
Molecular structure of isolate 1 (Apigenin).

The IHD value was calculated to be 11, which supports a structure containing three rings and eight additional degrees of unsaturation, consistent with a flavonoid backbone ( Figure 8).

### Molecular docking analysis of apigenin with granzyme B, perforin, and interferon-γ

The docking results demonstrated that apigenin exhibited binding affinities of −5.56 kcal/mol with both granzyme B and perforin, and −4.95 kcal/mol with IFN-γ. These values were comparable to those of levamisole, which showed binding affinities of −5.08 kcal/mol (granzyme B), −5.36 kcal/mol (perforin), and −4.82 kcal/mol (IFN-γ) ( Table 2). In terms of molecular interactions, apigenin formed hydrogen bonding with His151 in granzyme B, and additional interactions including Pi-alkyl (Leu39, Lys40), sulfur-X (Lys149), and van der Waals (Phe57). For perforin, interactions included Pi-alkyl (Ala8, Leu11, Ile73), Pi-Pi stacking (Tyr53), and Pi-sulfur (Met77). For IFN-γ, apigenin exhibited multiple hydrogen bonds (Arg143, Arg372, Gln374, Asp544), Pi-alkyl interaction (Pro546), and ionic interactions including Pi-cation/anion (Arg378, Gly551). Levamisole, in comparison, exhibited hydrogen bonding with Ser195 (granzyme B), Arg372 (IFN-γ), and various hydrophobic and Pi-based interactions with all three proteins ( Table 3).

**Table 2.** Binding affinities of apigenin with granzyme B, perforin, and IFN-γ.

No	Binding affinity (kcal/mol)		
	Granzyme B	Interferon-γ	Perforin
Apigenin	-5.56	-4.95	-5.56
Levamisole	-5.08	-4.82	-5.36

**Table 3.** Binding interaction of apigenin with granzyme B, perforin, and IFN-γ.

Name	3D interaction	2D interaction
Apigenin Perforin	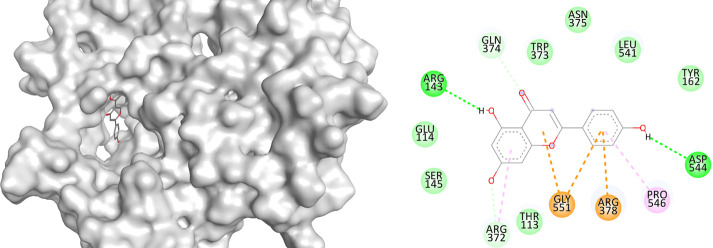	
Apigenin Interferon-γ	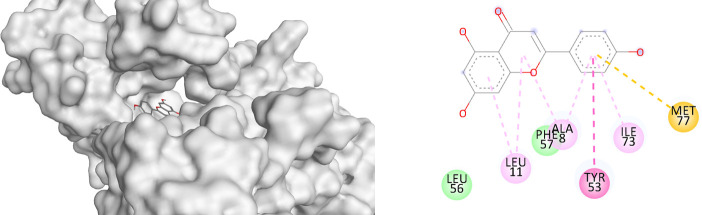	
Apigenin Granzyme B	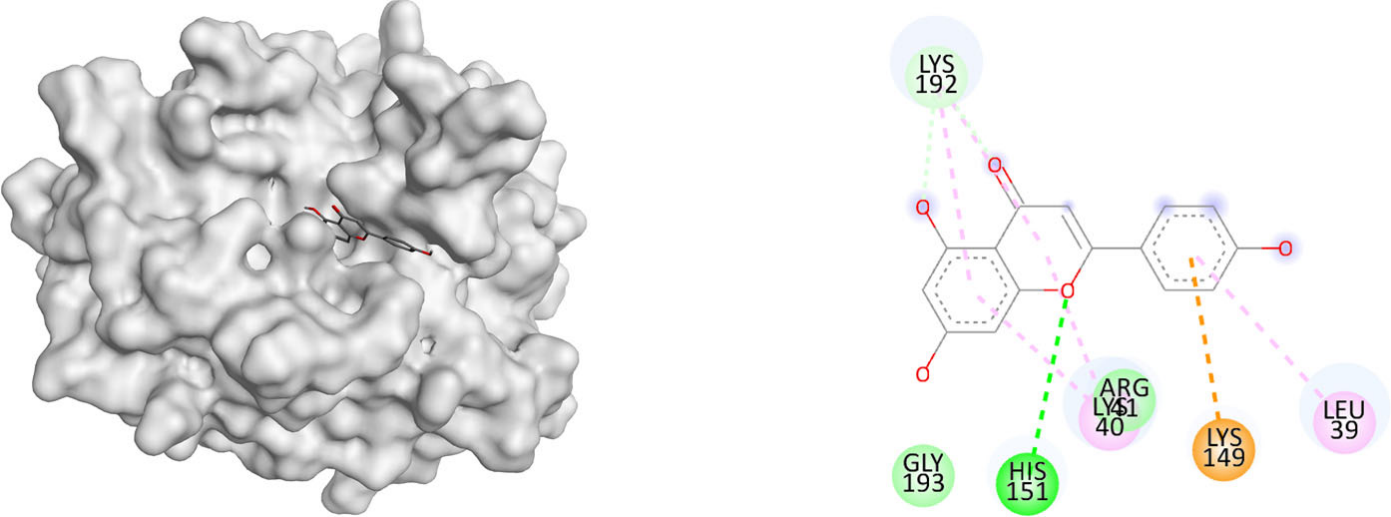	

### Immunostimulatory effects of apigenin on granzyme B, Perforin, and Interferon-γ expression in mice induced with COVID-19 vaccine

**Figure 9.**
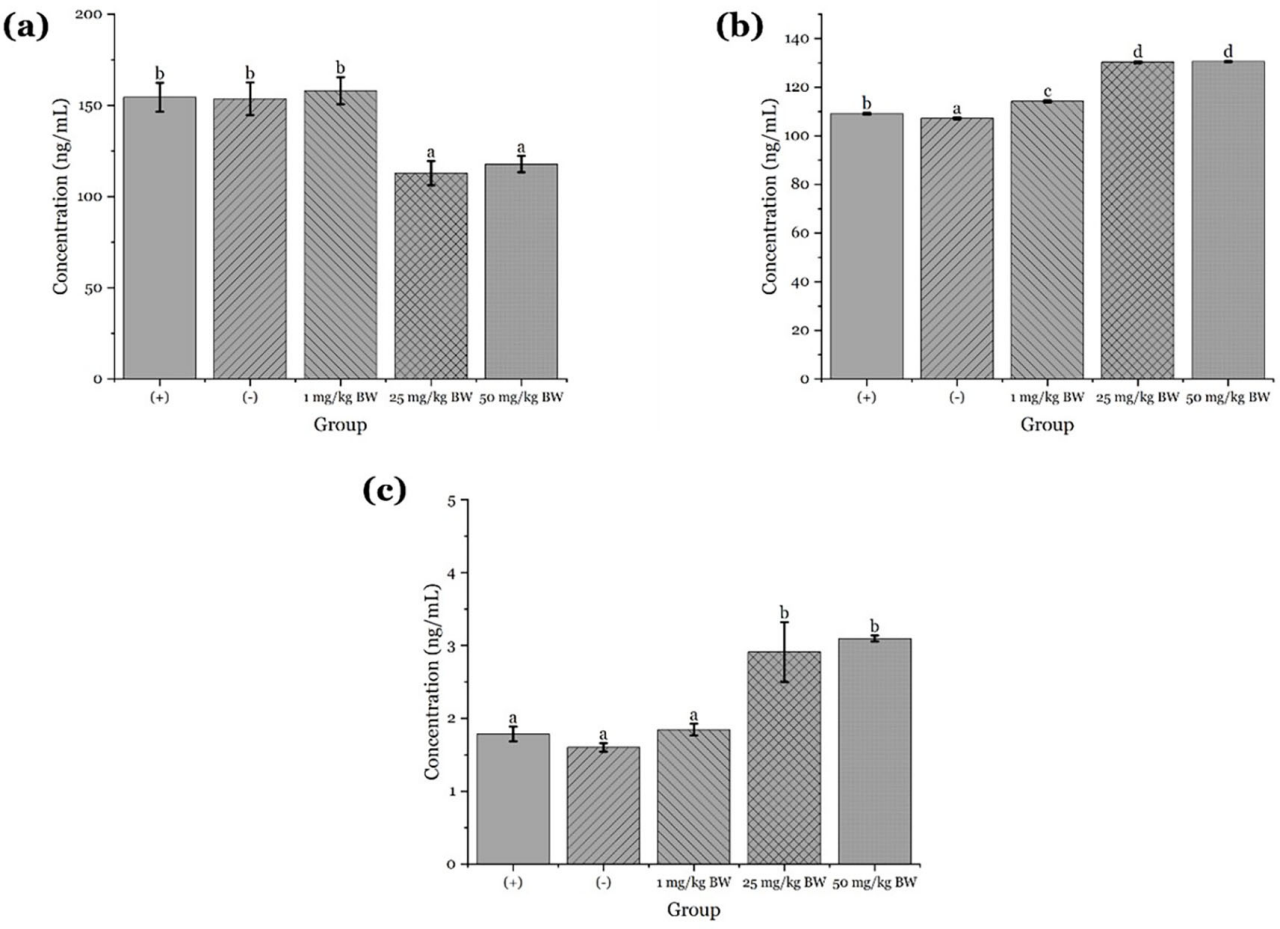
The effect of apigenin administration on the concentrations of: (a) granzyme; (b) interferon-γ; and (c) perforin. Different letters on the graphs indicate significant differences (P < 0.05) based on Duncan's test.

The administration of apigenin isolated from *Peronema canescens* (sungkai) leaves was evaluated for its immunostimulant effects in male white mice induced with the COVID-19 vaccine. The study involved five experimental groups: a negative control group receiving 0.5% NaCMC, a positive control group receiving the COVID-19 vaccine, and three treatment groups receiving apigenin at doses of 1 mg/kgBW, 25 mg/kgBW, and 50 mg/kgBW, respectively. The ELISA results indicated a dose-dependent increase in granzyme B levels across the groups. The negative and positive control groups showed relatively lower granzyme B levels, with mean values of 150.83 and 151.98, respectively. In contrast, the treatment groups demonstrated higher values, reaching 160.75 at 1 mg/kgBW, 161.38 at 25 mg/kgBW, and 162.09 at 50 mg/kgBW ( Figure 9a). Statistical analysis using one-way ANOVA confirmed significant differences among the groups (p < 0.05), and Duncan’s post hoc test revealed that all treatment groups formed a separate subset from the controls, indicating a significant stimulatory effect of apigenin on granzyme B production.

Similarly, perforin levels showed a notable increase with escalating doses of apigenin. The negative and positive control groups had lower mean values of 1,602 and 1,789, respectively, whereas the treatment groups exhibited higher values of 1,846 at 1 mg/kgBW, 2,917 at 25 mg/kgBW, and 3,086 at 50 mg/kgBW ( Figure 9b). Statistical validation through one-way ANOVA revealed a significant increase in perforin levels (p < 0.05), and Duncan’s test indicated a consistent trend with the granzyme B data, supporting the conclusion that apigenin administration enhances perforin expression.

For interferon-γ, the ELISA data also demonstrated a dose-dependent increase. The negative and positive control groups exhibited mean levels of 107.18 and 109.10, respectively, while the treatment groups showed elevated levels of 114.20, 130.32, and 130.57 for doses of 1, 25, and 50 mg/kgBW, respectively ( Figure 9c). Although body weight was not significantly different among the groups, interferon-γ levels increased significantly with higher doses of apigenin. Normality testing with the Shapiro-Wilk test (p > 0.05) and homogeneity testing (p > 0.05) confirmed the appropriateness of one-way ANOVA, followed by Duncan’s test, which indicated the highest stimulation occurred at the 50 mg/kgBW dose.

## Discussion

The comprehensive characterization of the isolate from *Peronema canescens* (sungkai) leaves using spectroscopic and spectrometric techniques confirmed its identity as apigenin, a well-known flavonoid. The UV-Vis spectral data revealed the presence of a conjugated chromophore system typical of flavonoids, ^24^ while FTIR spectra confirmed the existence of phenolic hydroxyl groups and a conjugated carbonyl moiety. Further structural elucidation using ^1^H-NMR and ^13^C-NMR indicated the presence of a flavone backbone, with specific proton and carbon signals corresponding to methine and quaternary carbons within an aromatic system. ^25, 26^ DEPT-135 and HSQC spectra allowed the differentiation of protonated from non-protonated carbons, while COSY and HMBC spectra elucidated substitution patterns on the flavone rings, confirming ortho and meta relationships and assigning the phenolic hydroxyl groups to C-5, C-7, and the para-position on the B-ring. Mass spectrometry validated the molecular formula (C _15_H _10_O _5_) and molecular weight (268.992), consistent with the known structure of apigenin. The Index of Hydrogen Deficiency (IHD) of 11 further supported the proposed flavonoid structure, composed of three aromatic rings and multiple degrees of unsaturation. These findings are consistent with those of a previous study on the apigenin compound in Gentiana veitchiorum. ^26^ Based on all this data, the compound was identified as 5,7-dihydroxy-2-(4-hydroxyphenyl)-4H-chromen-4-one, commonly known as apigenin.

The molecular docking results support the hypothesis that apigenin possesses immunomodulatory potential by interacting with key effector proteins involved in cytotoxic immune responses. The relatively low binding affinity of apigenin toward granzyme B and perforin (−5.56 kcal/mol) suggests a possible stabilizing interaction that may influence their expression or activity in vivo. This finding is in line with the in vivo results, which demonstrate a dose-dependent increase in the expression of these proteins following apigenin administration. Notably, apigenin showed multiple interaction types with interferon-γ, including hydrogen bonds and ionic contacts. The presence of multiple stabilizing interactions (e.g., Arg143, Arg372, Gln374) suggests that apigenin may play a role in enhancing or modulating IFN-γ signaling pathways. In comparison with levamisole, a known immunostimulant, apigenin showed similar or slightly better docking scores and comparable interaction patterns across the three proteins. This suggests that apigenin may exert its effects through a mechanism partially overlapping with levamisole, yet possibly more selective or synergistic in enhancing cytotoxic immune responses without broadly modulating other inflammatory mediators, such as IL-10. The molecular interactions observed, including hydrogen bonding, Pi-alkyl stacking, and electrostatic forces, further reinforce the specificity of apigenin toward cytotoxic effector proteins. These findings are consistent with previous reports describing the ability of flavonoids to modulate T cell and NK cell function through direct or indirect modulation of granzyme B and perforin expression. ^27, 28^

The in vivo findings of this study further confirm that apigenin isolated from sungkai leaves possesses potent immunostimulatory activity. The observed increases in granzyme B, perforin, and interferon-γ levels in apigenin-treated mice support its role in enhancing cellular immune responses. The significant elevation in granzyme B levels aligns with previous studies reporting that apigenin can upregulate granzyme B expression through the activation of the JNK and ERK signaling pathways, thereby enhancing the cytotoxic function of natural killer (NK) cells, particularly in their response to tumors or virally infected cells. ^29, 30^ Granzyme B and perforin are crucial cytolytic proteins secreted by cytotoxic T lymphocytes (CTLs) and NK cells to induce apoptosis in infected or malignant cells, and the marked increase in their levels in this study highlights apigenin’s capacity to potentiate the immune defense mechanisms of the host. ^31^

Furthermore, the elevation of interferon-γ levels suggests that both innate and adaptive immune responses are enhanced. Interferon-γ is a pivotal cytokine produced predominantly by activated T cells and NK cells and plays a central role in orchestrating the immune response against viral infections. ^32^ The increasing trend in interferon-γ production across the treatment groups suggests that apigenin enhances cytokine-mediated signaling, which is crucial for the activation and proliferation of immune effector cells. These results are in agreement with the existing literature, which suggests that flavonoids, including apigenin, exert immunomodulatory effects through their antioxidant properties and interactions with immune signaling pathways. ^32^

The presence of other phytochemical constituents in sungkai leaves, such as tannins, alkaloids, and phenolics, may further contribute to the immunostimulatory potential observed. However, among these, apigenin appears to be the most bioactive compound. Previous research has demonstrated that apigenin can enhance NK cell activity by modulating the expression of key effector molecules. ^33^ This was also evidenced by a previous study that highlighted the importance of hydroxylation patterns in the flavone structure, which influence the immunostimulatory potency of such compounds. Additionally, a previous study demonstrated that total flavonoids from *Hippophae rhamnoides* can enhance NK cell cytotoxicity by upregulating the expression of granzyme and perforin, which further supports the proposed mechanism of action for apigenin. ^33^

## Conclusion

Sungkai ( *Peronema canescens* Jack.) leaves are known to contain various secondary metabolites, among which flavonoids, particularly apigenin, have shown promising immunostimulatory potential. In this study, Isolate 1 obtained from the ethyl acetate extract was identified as apigenin through comprehensive spectroscopic and spectrometric analysis. Molecular docking simulations were performed to explore the potential immunomodulatory mechanism of apigenin against granzyme B, perforin, and interferon-γ (IFN-γ). Subsequent *in vivo* assays in mice demonstrated that apigenin, administered at doses of 1 mg/kg BW, 25 mg/kg BW, and 50 mg/kg BW, significantly enhanced the expression of key immune effector molecules—granzyme B, perforin, and interferon-γ—which play critical roles in cellular immune responses.

Taken together, the integrated chemical, computational, and biological findings confirm that apigenin isolated from sungkai leaves possesses potent immunostimulant and anti-inflammatory properties. Its ability to modulate immune signaling pathways and enhance the expression of cytolytic proteins highlights its potential as a natural therapeutic agent. These results support further investigation and development of apigenin as an immune-enhancing and antiviral compound, especially for applications in immunocompromised individuals or as an adjuvant in vaccine formulations.

## Ethical considerations

This study has obtained approval from the code of ethics for health research, Faculty of Pharmacy, Andalas University with the number: 93/UN16.10.D.KEPK-FF/2024.

## Underlying data

**Zenodo:** Isolation of Apigenin from Sungkai ( *Peronema canescens*) Leaves and Its Immunomodulatory Effects: An in vivo Study on Granzyme B, Interferon-γ, and Perforin Expression with Supporting In Silico Analysis. Doi: 10.5281/zenodo.15980341 ^34^

This project contains the following underlying data:

- ARRIVE Checklist
- Data Granzyme
- Data Interferon-γ
- Data Perforin

## Reporting guidelines

**ARRIVE checklist** for “Isolation of Apigenin from Sungkai ( *Peronema canescens*) Leaves and Its Immunomodulatory Effects: An in vivo Study on Granzyme B, Interferon-γ, and Perforin Expression with Supporting In Silico Analysis”, Doi: 10.5281/zenodo.15980341 ^34^

## Data Availability

Isolation of Apigenin from Sungkai (Peronema canescens) Leaves and Its Immunomodulatory Effects: An in vivo Study on Granzyme B, Interferon-γ, and Perforin Expression with Supporting In Silico Analysis © 2025 by Dwisari Dillasamola, M.Farm, Apt is licensed under Creative Commons Attribution 4.0 International.
