# Supplementary material for: Isolation of Apigenin from Sungkai ( Peronema canescens) Leaves and Its Immunomodulatory Effects: An In Vivo Study on Granzyme B, Interferon-γ, and Perforin Expression with Supporting In Silico Analysis
Source: F1000Res. 2025 Aug 8;14:774. [Version 1] doi: 10.12688/f1000research.167153.1 (PMC12481153; doi:10.12688/f1000research.167153.1)
Supplement: Peer review history

# Reviewer report: Reviewer response for version 1

**DOI:** 10.5256/f1000research.184242.r411394

**Published:** 2025-10-07

**Authors:** Pitchaporn Wanyo

**Affiliations:** 1 Kalasin University, Kalasin, Kalasin, Thailand

The manuscript is scientifically relevant and contributes to the understanding of the immunomodulatory properties of Peronema canescens (sungkai) and its flavonoid apigenin. The integration of in silico docking with in vivo immunological evaluation is commendable. However, several areas require clarification, refinement of experimental justification, and improvement in scientific presentation and discussion depth.

**1. Abstract**

 The Abstract should emphasize the novelty of isolating apigenin, integration of in silico–in vivo design, and key quantitative findings.

**2. Introduction**

 2.1 Clarify the research gap—specifically, that apigenin’s immunostimulatory role from Peronema canescens has not been previously demonstrated.

 2.2 Add recent (2023–2025) references about apigenin and flavonoid-based immunomodulators.

 2.3 Justify selection of granzyme B, perforin, and IFN-γ as immune markers representing cytotoxic activity.

**3. Materials and Methods**

 3.1 Reduce redundancy in brand and catalog details; focus on replicability.

 3.2 Clarify animal randomization, blinding, and justification for sample size (n=5).

 3.3 Explain rationale for using COVID-19 vaccine as an immune activator in mice, and provide ethical justification.

 3.4 Include an apigenin-only control group to assess independent effects.

 3.5 Mention toxicity or LD50 data or cite prior literature confirming safety at tested doses.

 3.6 Include effect sizes or confidence intervals for ANOVA results.

**4. Results**

 4.1 Summarize spectroscopic data in a single table or composite figure.

 4.2 Report RMSD, hydrogen bonds, and binding pocket residues in docking results.

 4.3 Improve figure resolution, axis labeling, and statistical annotations.

 4.4 Discuss biological relevance of small increases (≈5–20%) in immune markers.

**5. Discussion**

 5.1 Focus more on mechanism—describe apigenin’s modulation of JNK/ERK or NF-κB signaling pathways.

 5.2 Discuss study limitations: lack of interleukin profiling, no apigenin-only control, and untested toxicity.

 5.3 Compare results with other flavonoid immunomodulators (quercetin, luteolin).

 5.4 Include recent references (2024–2025) to enhance depth.

**6. Conclusion**

 6.1 Rephrase to indicate potential immunostimulatory effects rather than definitive proof.

 6.2 Suggest future work: cytokine profiling (IL-2, IL-10), NK cell assays, or mechanistic pathway analysis.

Is the work clearly and accurately presented and does it cite the current literature?

Yes

If applicable, is the statistical analysis and its interpretation appropriate?

Yes

Are all the source data underlying the results available to ensure full reproducibility?

Partly

Is the study design appropriate and is the work technically sound?

Yes

Are the conclusions drawn adequately supported by the results?

Yes

Are sufficient details of methods and analysis provided to allow replication by others?

Partly

Reviewer Expertise:

Natural products, bioactive compounds

I confirm that I have read this submission and believe that I have an appropriate level of expertise to confirm that it is of an acceptable scientific standard, however I have significant reservations, as outlined above.

# Reviewer report: Reviewer response for version 1

**DOI:** 10.5256/f1000research.184242.r411397

**Published:** 2025-09-29

**Authors:** Bushra Hussain Shnawa

**Affiliations:** 1 Soran University, Kurdistan, Kurdistan, Iraq

The manuscript is novel, well organized and  designed, as well as well written. However,  minor comments need to be addressed.

 1- What about the toxicity of the apigenin, which is classified as a flavonoid, to determine its LD50?

 2- The authors didn't use another standard immunostimulatory material to compare with it. To determine the levels of the immune stimulation of apigenin in comparison with others.

 3- The proposed mechanisms of the immunostimulatory potential of apigenin need to be presented in a schematic diagram.

 4- Why did the authors select granzyme B, perforin, and interferon-γ (IFN-γ)? No interleukin included.

 Good luck

 .

Is the work clearly and accurately presented and does it cite the current literature?

Yes

If applicable, is the statistical analysis and its interpretation appropriate?

Not applicable

Are all the source data underlying the results available to ensure full reproducibility?

Yes

Is the study design appropriate and is the work technically sound?

Yes

Are the conclusions drawn adequately supported by the results?

Yes

Are sufficient details of methods and analysis provided to allow replication by others?

Yes

Reviewer Expertise:

Medical biosciences, Immunology, Medical parasitology

I confirm that I have read this submission and believe that I have an appropriate level of expertise to confirm that it is of an acceptable scientific standard.

# Reviewer report: Reviewer response for version 1

**DOI:** 10.5256/f1000research.184242.r411398

**Published:** 2025-09-22

**Authors:** Valentina Mittova

**Affiliations:** 1 University Geomedi, Tbilisi, Tbilisi, Georgia

The article presents interesting results on the isolation of apigenin from plant material and its immunostimulatory effect. However, more substantiation should be given to experiments with the administration of apigenin.

 1. Why was the COVID-19 vaccine chosen as an immunostimulator?

 2. Why was the control group, which received apigenin and was not vaccinated, not used in the experiments?

 3. In Fig. 9 signs like "+' and "-" should be explained.

 4. The conclusion section should be rephrased; the statement "apigenin significantly enhanced the expression of key immune effector molecules—granzyme B, perforin, and interferon-γ" is not accurate, since it was revealed only in preliminary vaccinated mice.

Is the work clearly and accurately presented and does it cite the current literature?

Yes

If applicable, is the statistical analysis and its interpretation appropriate?

Yes

Are all the source data underlying the results available to ensure full reproducibility?

Partly

Is the study design appropriate and is the work technically sound?

Yes

Are the conclusions drawn adequately supported by the results?

Yes

Are sufficient details of methods and analysis provided to allow replication by others?

Partly

Reviewer Expertise:

Antioxidant and antibacterial properties of medicinal plants.

I confirm that I have read this submission and believe that I have an appropriate level of expertise to confirm that it is of an acceptable scientific standard, however I have significant reservations, as outlined above.

# Reviewer report: Reviewer response for version 1

**DOI:** 10.5256/f1000research.184242.r405596

**Published:** 2025-09-16

**Authors:** Dharmalingam Kirubakaran

**Affiliations:** 1 Saveetha Institute of Medical and Technical Sciences, Chennai, Tamilnadu, India

The manuscript titled *"* Isolation of Apigenin from Sungkai (Peronema canescens) Leaves and Its Immunomodulatory Effects: An In Vivo Study on Granzyme B, Interferon-γ, and Perforin Expression with Supporting In Silico Analysis *"* presents a study with strong potential and scientific relevance. However, following a thorough evaluation, I recommend minor revision and after acceptance for publication

1. Revise grammar throughout the manuscript and correct all errors.
2. Strengthen and improve the Discussion section.
3. Rewrite the Abstract for clarity and refine the Conclusion.
4. Ensure all figures are of high quality and appropriately corrected.
5.  Overall, the manuscript is good, but it requires the addition of recent references and a thorough grammar check across all sections.

- We have relevant references that can be cited to improve the Discussion section. ( refer to 1,2,3,4)

Is the work clearly and accurately presented and does it cite the current literature?

Yes

If applicable, is the statistical analysis and its interpretation appropriate?

No

Are all the source data underlying the results available to ensure full reproducibility?

Yes

Is the study design appropriate and is the work technically sound?

Yes

Are the conclusions drawn adequately supported by the results?

Yes

Are sufficient details of methods and analysis provided to allow replication by others?

Yes

Reviewer Expertise:

Good data adn research

I confirm that I have read this submission and believe that I have an appropriate level of expertise to confirm that it is of an acceptable scientific standard.

## References

[1] : Radiolabeled nanoparticles in multimodal nuclear imaging, diagnostics and therapy. *Journal of Radioanalytical and Nuclear Chemistry* .2025;334(7) : 10.1007/s10967-025-10233-9 4403-4418 10.1007/s10967-025-10233-9

[2] https://www.scopus.com/pages/publications/84864012845.

[3] : Sunlight-driven photocatalytic and anticancer properties of biogenic synthesized gold nanoparticles (AuNPs) employing Polygala elongata. *Journal of King Saud University - Science* .2024;36(5) : 10.1016/j.jksus.2024.103158 10.1016/j.jksus.2024.103158

[4] https://www.scopus.com/pages/publications/77956130065.
